# LEF1, TFE3, and AR are putative diagnostic markers of solid pseudopapillary neoplasms

**DOI:** 10.18632/oncotarget.21854

**Published:** 2017-10-16

**Authors:** Eun Kyung Kim, Mi Jang, Minhee Park, Hoguen Kim

**Affiliations:** ^1^ Department of Pathology, Yonsei University College of Medicine, Seoul, Republic of Korea; ^2^ Departments of Pathology and Brain, Korea 21 PLUS Project for Medical Science, Yonsei University College of Medicine, Seoul, Republic of Korea; ^3^ Healthcare Review and Assessment Committee, Health Insurance Review & Assessment Service, Seoul, Republic of Korea

**Keywords:** solid pseudopapillary neoplasm, lymphoid enhancer-binding factor 1, transcription factor for immunoglobulin heavy-chain enhancer 3, androgen receptor, Ki-67, Pathology Section

## Abstract

The diagnosis of solid pseudopapillary neoplasms (SPNs) is challenging because some SPNs share many similar morphological and immunohistochemical features with other pancreatic neoplasms. In this study, we investigated potential diagnostic markers of SPN.

Based on the SPN-specific upregulated genes from a previous DNA microarray and proteome study, we selected six immunohistochemical markers [beta-catenin, androgen receptor (AR), lymphoid enhancer-binding factor 1 (LEF1), transcription factor for immunoglobulin heavy-chain enhancer 3 (TFE3), fused in sarcoma (FUS), and WNT inhibitory factor 1 (WIF-1)]. We also evaluated the Ki-67 proliferative index to investigate its associations with prognosis. To validate these markers, we studied 91 SPNs as well as 51 pancreatic ductal carcinomas (PDC) and 48 neuroendocrine tumors (NET) as controls.

We found frequent and diffuse nuclear expressions of β-catenin (98.9%), AR (81.3%), LEF1 (93.4%), TFE3 (74.7%), FUS (84.6%), and cytoplasmic expression of WIF-1 (96.7%) in SPNs. In contrast, PDCs and NETs showed no expression. (*P* < 0.001). When beta-catenin, LEF1, and TFE3 staining were combined, the sensitivity and specificity were 100% and 91.9%, respectively. Four (4.4%) SPNs showed distant metastasis and these tumors were associated with a relatively high Ki-67 proliferative index (≥ 5%; *P* = 0.013).

We identified LEF1, TFE3, and AR as putative diagnostic markers of SPN, auxiliary to β-catenin. Incorporated into an immunohistochemical panel, these markers could be beneficial to distinguish SPN from PDC and NET. In addition, we suggest that the Ki-67 proliferative index can be a predictive marker of metastasis in SPNs.

## INTRODUCTION

Among pancreatic tumors, solid pseudopapillary neoplasm (SPN) is very rare and comprises only 1–3% of primary pancreatic tumors. In spite of its clinical rarity, the clinical and pathologic findings are well recognized. SPN is composed of poorly cohesive monomorphic epithelial cells that form solid, pseudopapillary structures, and it occurs predominantly in young women [1].

SPNs are genetically characterized by somatic mutations in exon 3 of *CTNNB1*, which encodes β-catenin [2, 3]. In addition, the molecular regulatory networks of SPN have recently been well-characterized [4-7]. Through an integrative analysis of mRNA, microRNA, and proteome expression profiles, we have identified many (> 1,000) SPN-specific upregulated genes, specifically activated Wnt/β-catenin, Hedgehog, and androgen receptor (AR) signaling pathways [6, 8].

However, there are several unresolved issues in SPNs. First, putative diagnostic immunohistochemical markers are necessary. The pathologic diagnosis of SPNs is usually made using a combination of histologic and immunohistochemical markers, including beta-catenin, CD10, chromogranin, and E-cadherin [1, 9, 10]. Diffuse cytoplasmic and nuclear localization of β-catenin is almost always found in SPN, and these findings are helpful in the diagnosis of SPNs. Nevertheless, some SPNs are histologically and immunohistochemically similar to pancreatic neuroendocrine tumors (NETs), and nuclear localization of β-catenin can also be found in some pancreatic ductal adenocarcinomas (PDCs) and NETs. Second, there are still no definite prognostic and diagnostic criteria for predicting the malignant behavior of SPNs. Although most SPNs have low malignant potential and can be cured using surgical resection [11-13], it has been reported that up to 20% of SPNs showed recurrence or distant metastasis [11-16].

In this study, we selected several immunohistochemical markers from the SPN-specific up-regulated genes and validated their diagnostic usability in SPNs. Additionally, we investigated clinicopathologic parameters associated with the malignant behavior of SPNs.

## RESULTS

### Clinicopathologic characteristics of SPN

**Figure 1:**
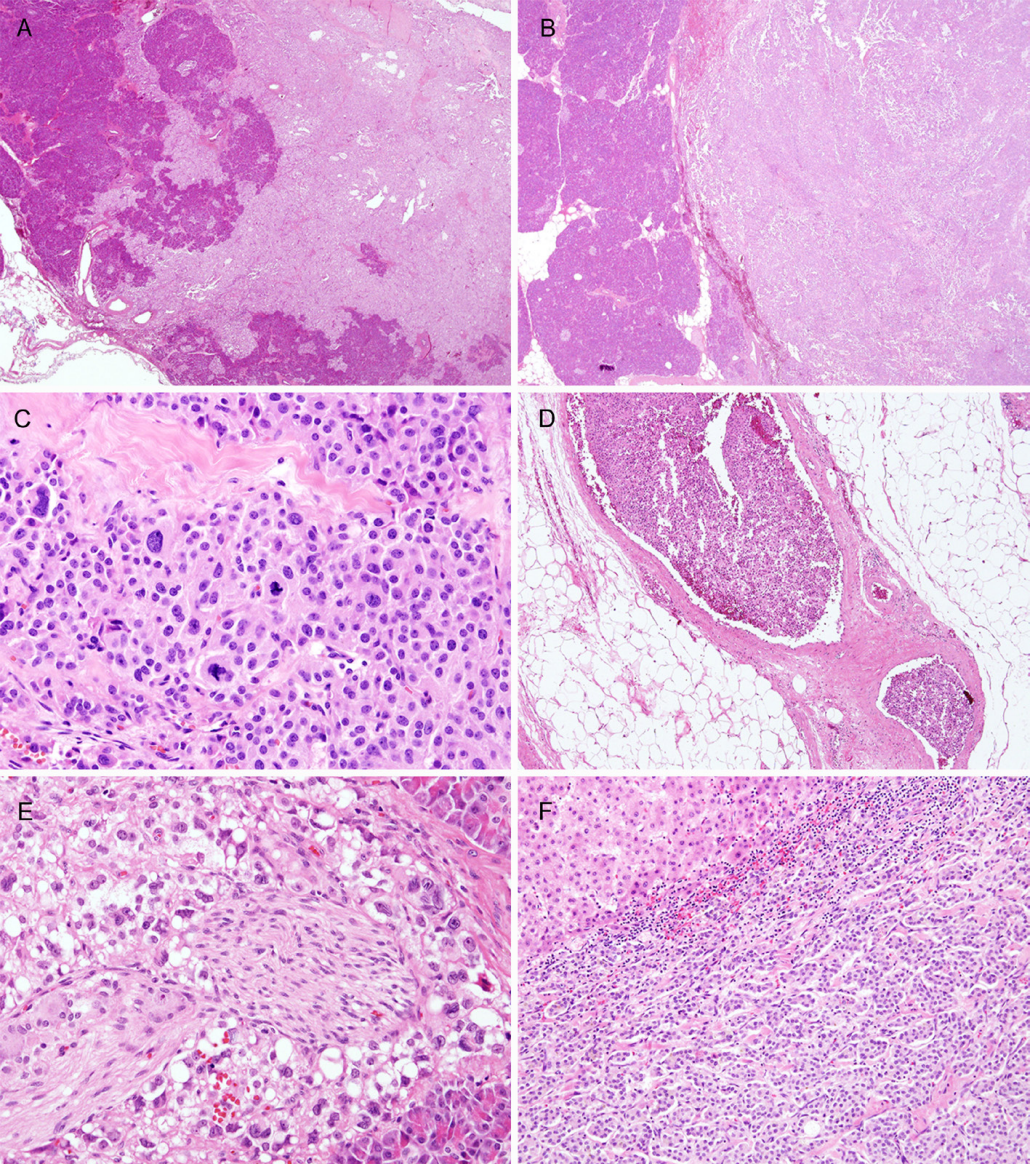
Histologic features (H & E stain) of SPNs **A**. Infiltrative and **B**. expanding border of tumor (A and B, each original magnification x12.5). **C**. Pleomorphism and mitosis (original magnification x100). **D**. Lymphovascular invasion (original magnification x40). E. Perineural invasion (original magnification x100). F. Liver metastasis (original magnification x40).

The basic clinicopathologic findings are listed in Table 1 and Figure 1. Sixty-one patients (67.0%) were ≤ 40 years old (median, 34 years old; range, 8–67 years), with a predominance of women (75, 82.4%). The tumor size varied from 1 to 16 cm (average, 4.7 cm). Six (6.6%) tumors measured more than 10 cm. More than 80% of the tumors had at least a focally infiltrative border (73; 80.2%, Figure 1A) and 40 (44%) showed peripancreatic adipose tissue invasion. Cellular pleomorphism (Figure 1C) was observed in 6 (6.6%) cases and mitoses were found in up to 2/10 high-power fields (HPFs) in 4 (4.3%) cases. Calcification and extensive hemorrhage/necrosis were present in 38 (43.6%) and 25 (27.5%) of tumors, respectively. Small cytoplasmic hyaline globules, foamy histiocytes, cholesterol clefts, and myxoid stromal changes were also observed. Three cases (3.3%) showed lymphovascular invasion (Figure 1D) and lymph nodes metastasis, respectively. Perineural invasion (Figure 1E) was a relatively frequent finding in 47 (51.6%) cases. Distant metastasis occurred in 4 (4.4%, Figure1F) patients and recurrence developed in 3 (3.3%) patients.

**Table 1:** Clinicopathologic characteristics of 91 SPNs

		Tumor border		
	Total, n (%)	Expanding	Infiltrative	*P*-value
	91 (100)	18 (19.8)	73 (80.2)	
Sex				*0.035*
Male	16 (17.6)	0 (0)	16 (21.9)	
Female	75 (82.4)	18 (100)	57 (78.1)	
Age (y)				*0.160*
≤ 40	61 (67.0)	15 (83.3)	46 (63.0)	
> 40	30 (33.0)	3 (16.7)	27 (37.0)	
Size (cm)				*0.001*
0-5	58 (63.7)	5 (27.8)	53 (72.6)	
5.1-10	27 (29.7)	11 (61.1)	16 (21.9)	
>10.1	6 (6.6)	2 (11.1)	4 (5.5)	
Extensive hemorrhage/necrosis	25 (27.5)	11 (61.1)	14 (19.2)	*0.001*
Pleomorphism	16 (17.6)	2 (11.1)	14 (19.2)	*0.730*
Lymphovascular invasion	3 (3.3)	0 (0)	3 (4.1)	*0.611*
Perineural invasion	47 (51.6)	5 (27.8)	42 (57.5)	*0.034*
Lymph node metastasis	3 (3.3)	0 (0)	3 (4.1)	*0.611*
Distant metastasis	4 (4.4)	0 (0)	4 (4.4)	*0.611*

The rim of most SPNs is known to be infiltrative. However, since some SPNs show totally expanding borders, we examined whether clinicopathologic parameters differ depending on whether the margin of tumor invades surrounding parenchyma. When the border of SPNs was divided into at least focally infiltrative pattern and totally expanding pattern, male sex (p = 0.035), smaller tumor size (p < 0.001), non-extensive hemorrhage and/or necrosis (p = 0.001), and frequent perineural invasion (p = 0.034) were found in the infiltrative SPNs (Table 1). We also found that SPNs in male patients had a lower incidence of hemorrhage and/or necrosis, more frequent infiltrative tumor borders, and peripancreatic invasion (Supplementary Table 2). They also showed a tendency toward more frequent lymph node metastasis (p = 0.079).

### Immunohistochemical expression in SPN, PDC, and NET

The results of the immunohistochemical analysis of 91 SPNs, 51 PDCs, and 48 NETs are shown in Table 2. Beta-catenin showed diffuse membranous expression in normal pancreatic tissue and AR was expressed in some of the normal pancreatic ductal epithelium. FUS and WIF-1 showed occasional staining in the cytoplasm of pancreatic ductal cells and acinar cells. LEF1 and TFE3 were not expressed in normal pancreatic tissue.

**Table 2:** Expression pattern of 6 markers in SPN

Protein	Expression site	SPN (%)	PDC (%)	NET (%)	*P*-value	Diagnosis of SPN	
						Sensitivity	Specificity
Beta-catenin	Nuclear	90 (98.9)	1 (2.0)	2 (4.2)	*<0.001*	98.9%	97.0%
AR	Nuclear	74 (81.3)	10 (19.6)	3 (6.3)	*<0.001*	81.3%	86.9%
LEF1	Nuclear	85 (93.4)	0 (0.0)	1 (2.1)	*<0.001*	93.4%	99.0%
TFE3	Nuclear	68 (74.7)	1 (2.0)	7 (14.6)	*<0.001*	74.7%	91.9%
FUS	Nuclear	77 (84.6)	32 (62.7)	15 (31.3)	*<0.001*	84.6%	52.5%
WIF-1	Cytoplasm	88 (96.7)	20 (39.2)	8 (16.7)	*<0.001*	96.7%	71.7%
Total		91 (100)	51 (100)	48 (100)			

**Figure 2:**
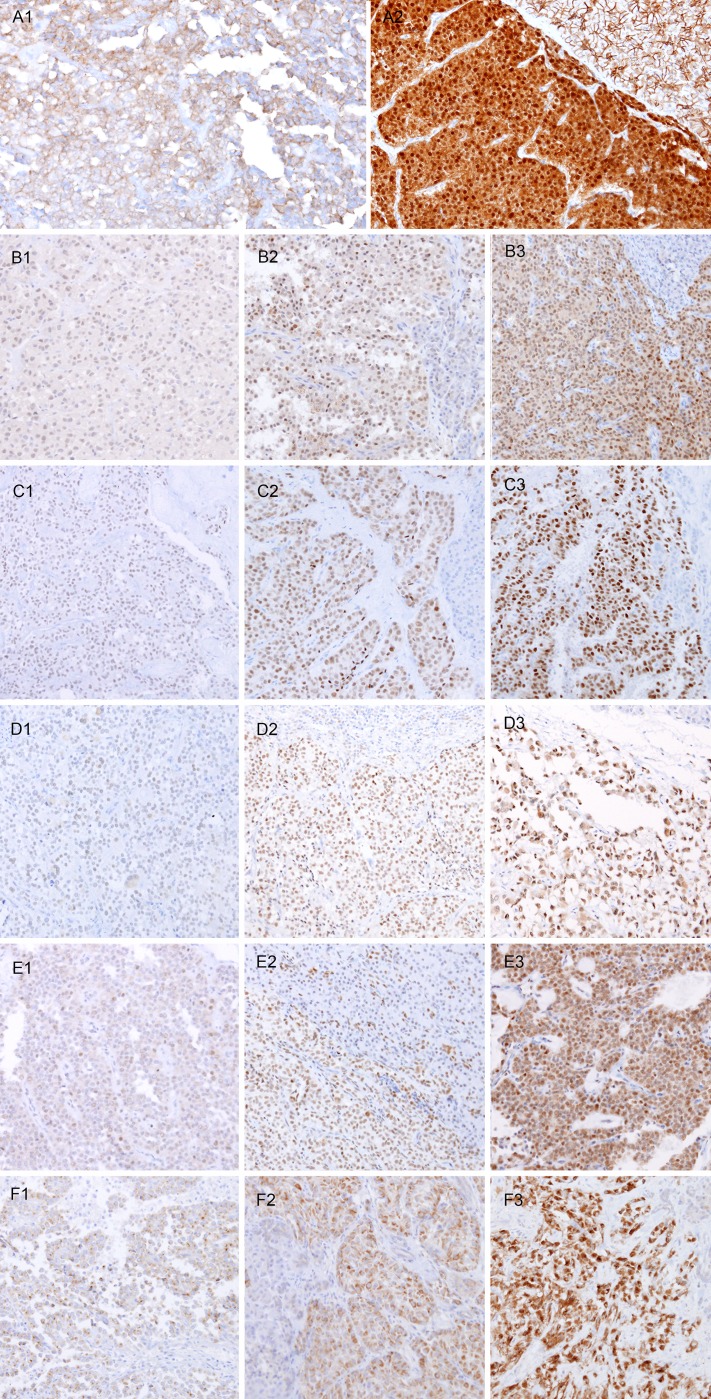
Representative immunohistochemical results of SPNs Beta-catenin (**A1**, negative and **A2**, strong positive), AR (**B1-B3**), LEF1 (**C1-C3**), TFE3 (**D1-D3**), and FUS (**E1-E3**) show nuclear expression. WIF-1 (**F1-F3**) show cytoplasmic expression. Sub-number indicates weak (1), moderate (2), and strong (3), respectively. Original magnification x100.

In the SPNs, nuclear expressions of β-catenin (98.9%), AR (81.3%), LEF1 (93.4%), TFE3 (74.7%), and FUS (84.6%), and cytoplasmic expression of WIF-1 (96.7%) were noted (Figure 2, p < 0.001). In contrast, PDCs and NETs showed no or occasional expression. Although some markers were positive in both SPNs and/or PDCs and NETs, there was no case showing strong expression of these markers in PDCs and NETs. Two of the 3 cases that had undergone decalcification during processing showed no expression of any of the markers, except for WIF-1 (weak) and β-catenin (moderate).

**Figure 3:**
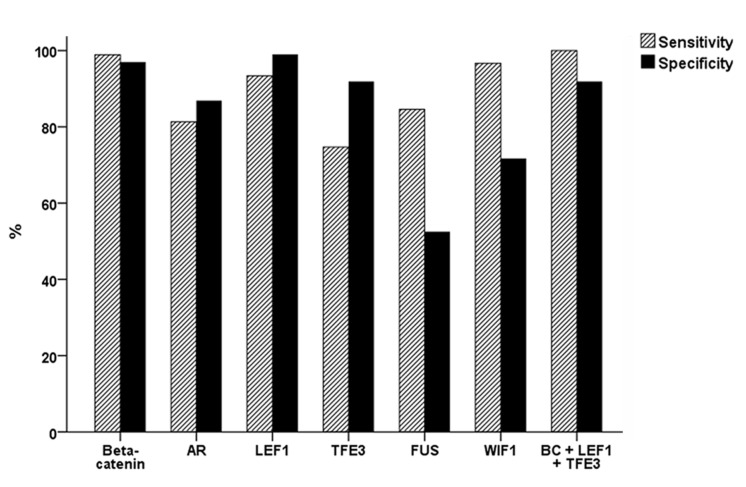
Sensitivity and specificity of six immunohistochemical markers for the diagnosis of SPN When beta-catenin, LEF1, and TFE3 are combined, the sensitivity and specificity are 100% and 91.9%, respectively.

After evaluating the diagnostic significance of these markers in pancreatic tumors, we described the sensitivity and specificity in Table 2 and Figure 3. Based on these findings, we concluded that LEF1, TFE3, and AR could be used as putative diagnostic markers of SPN. When β-catenin, LEF1, and TFE3 staining results were combined, the sensitivity and specificity were 100% and 91.9%, respectively.

We also evaluated the relationship between the clinicopathologic findings and the expression of these 6 markers. Among them, AR was more frequently expressed in male patients (p = 0.035). However, there were no other significant correlations between the expression of other markers and clinicopathologic parameters.

### A high Ki-67 proliferative index is associated with metastatic SPN

**Figure 4:**
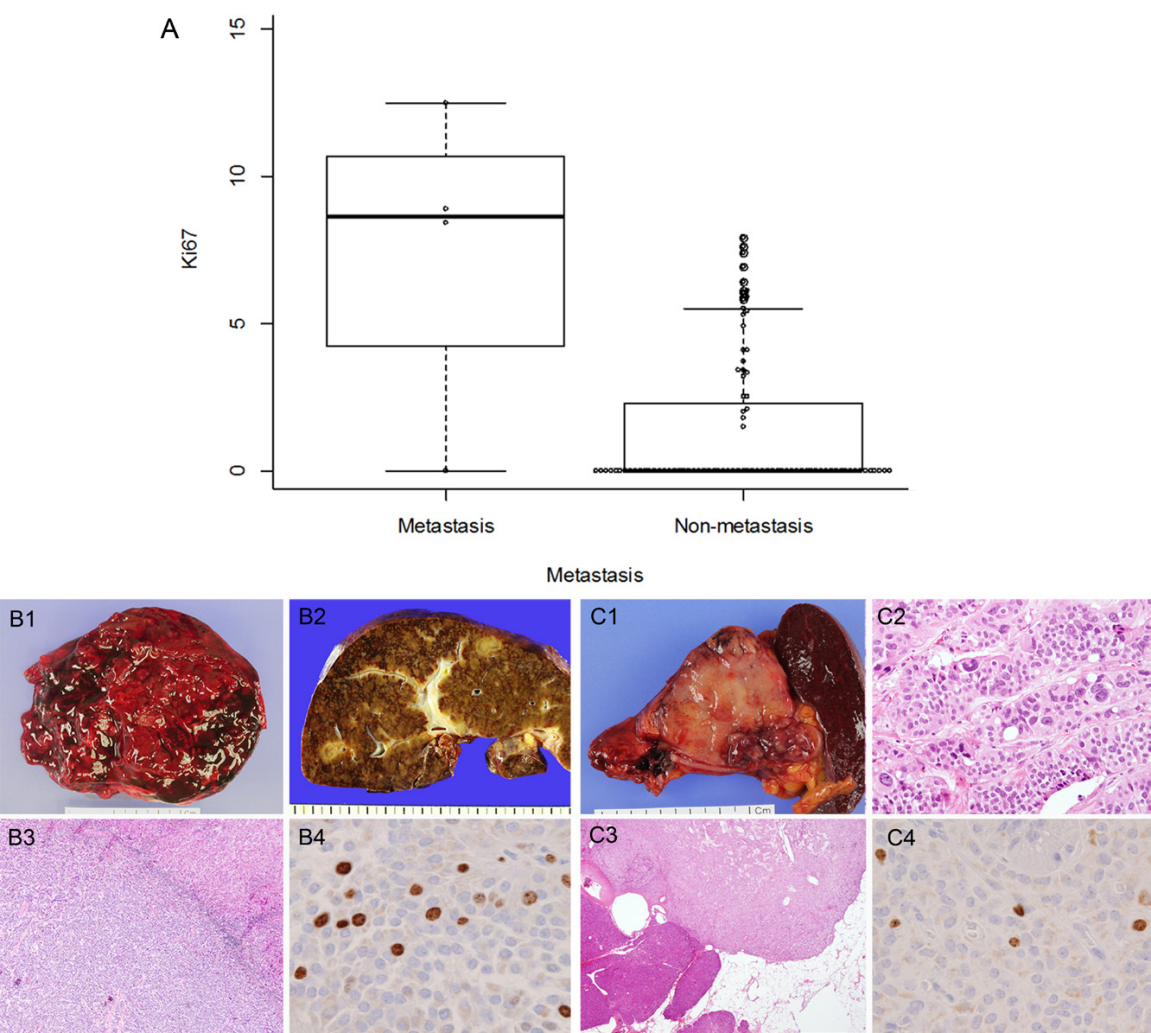
Ki-67 proliferative index and representative cases of SPNs with metastasis Ki-67 proliferative index is related to distant metastasis (**A**, *p =* 0.013). Gross features of primary SPN (**B1**) and metastatic SPN in the liver (**B2**). H & E appearances metastatic SPN (**B3**, original magnification x40) and relatively high Ki-67 proliferative index (8.9%; **B4**, original magnification x400). Gross features of non-metastatic SPN (**C1**) may have pleomorphism (**C2**, original magnification x100) or peripancreatic extension (**C3**, original magnification x40), but show relatively low Ki-67 proliferative index (1.5%; **C4**, original magnification x400).

In our SPN cohort, distant metastasis was noted in 4 (4.4%) cases. The sites of metastasis were the liver (3, 3.3%; Figure 4B) and peritoneum (1, 1.1%). The Ki-67 proliferative index was correlated with metastasis (p = 0.013, Table 3 and Figure 4A). Univariate logistic regression analysis revealed that lymphovascular invasion (OR = 21.75; p = 0.030), lymph node metastasis (OR = 22.50; p = 0.028), and a Ki-67 proliferative index of more than 5% (OR = 18.75; p = 0.014) were statistically significant risk factors for distant metastasis. The three recurred SPNs were associated with a larger tumor size (> 5cm, p = 0.043), as well as a Ki-67 proliferative index of more than 5% (p = 0.004). In univariate logistic regression analysis, lymphovascular invasion (OR = 44.00; p = 0.017) and lymph node metastasis (OR = 45.50; p = 0.011) were statistically significant risk factors for recurrence. However, nuclear pleomorphism (Figure 4C), mitotic count, perineural invasion, peripancreatic extension, and infiltrative tumor borders were not associated with distant metastasis or recurrence.

**Table 3:** Relationship between Ki-67 labeling index and metastasis/recurrence

	Ki-67 labeling index		
	0-5%	>5%	*P*-value
Metastasis, n (%)			*0.013*
Present	1 (25)	3 (75)	
Absent	75 (86.2)	12 (13.8)	
Recurrence, n (%)			*0.004*
Present	0 (0)	3 (100)	
Absent	76 (86.4)	12 (13.6)	

## DISCUSSION

At present, the pathologic diagnosis of SPN is based on histologic and immunohistochemical characteristics. SPN is composed of small monomorphic cells with clear or eosinophilic cytoplasm that form solid or pseudopapillary structures with poor cohesion. Mutations in exon 3 of the β-catenin gene have been identified in the majority of SPNs [2, 3] and diffuse nuclear expression of β-catenin is known to be a characteristic feature used in the diagnosis of SPN. Other diagnostic markers include E-cadherin (loss) [9, 17, 18], CD10 [19], alpha-1-antitrypsin [19], chromogranin (negative) [9, 16], CD99 (dot-like pattern) [20], beta form of estrogen receptors [21], galectin 3 [21], KIT [22], DOG1 [23], FLI-1 [24], and alpha-Methylacyl-CoAracemase (AMACR, P504S) [25]. However, a confirmatory diagnosis of SPN is still challenging because SPN shares many morphological and immunophenotypical features with other pancreatic tumors, especially PDCs and NETs. In this study, we aimed to identify additional and putative diagnostic markers of SPN based on upregulated genes analyzed using mRNA expression profiling [6] and availability of antibodies.

We demonstrated that the combination of several immunohistochemical markers enhanced the diagnostic accuracy of SPN. We initially selected 6 markers and found frequent and strong nuclear expression of beta-catenin, AR, LEF1, TFE3, and FUS, and cytoplasmic expression of WIF-1 in SPNs. All 6 markers showed expression differences between SPNs and other pancreatic tumors. Although some markers were positive in both SPNs and/or PDCs and NETs, strong expression of these markers was not found in any PDCs or NETs. Our results confirmed that beta-catenin is the most sensitive (98.9%) and specific (97%) marker for SPN. Additionally, we demonstrated that 3 putative markers (LEF1, TFE3, and AR) showed high rates of sensitivity and specificity. When beta-catenin, LEF1, and TFE3 were combined, the sensitivity and specificity were 100% and 91.9%, respectively. Based on these findings, we suggest that LEF1, TFE3, and AR are useful for the diagnosis of SPN, and the combination of these markers with beta-catenin is helpful to improve their sensitivity and specificity in the diagnosis of SPNs.

LEF1 is a member of the lymphoid enhancer-binding factor 1/T-cell factor (LEF1/TCF) complex [26]. It interacts with mutant *CTNNB1* and act as a regulator of the Wnt/CTNNB1 signaling pathway [26, 27]. It has been reported to be upregulated in SPNs compared with normal pancreatic tissue [6], PDCs, and NETs [7]. Singhi *et al.* [28] have demonstrated diffuse nuclear LEF1 overexpression in 27 SPNs with high sensitivity and specificity. We observed similar results in a larger cohort.

TFE3 is a member of the microphthalmia (MiT) family of transcription factors, which is composed of MITF, TFE3, TFEB, and TFEC. MiT transcription factors form homodimers or heterodimers that bind target promoters and act as regulators of melanocyte development, cellular proliferation, survival, motility, and metabolism, which are deregulated during oncogenic processes [29, 30]. The MITF/TFE3 family has also been shown to bind and enhance the activity of the LEF1 protein, and may promote propagation of Wnt signals in many cell types [31]. TFE3 also had been reported to interact with AR and FUS in SPNs based on gene regulatory network analysis, and it has also been suggested as a novel biomarker of SPNs [7].

AR is associated with cell cycle regulation and with the AR signaling pathway, which is activated in SPN [6, 7]. Moreover, codon changes and codon deletion mutations in AR have been found using whole exome sequencing, which also reveals its close relationship with CTNNB1 signaling [32]. Recent studies have aimed to identify gene regulatory networks in SPN and have found that CTNNB1, LEF1, AR, and TFE3 interact with each other by diverse pathways [6, 7]. These findings indicate that CTNNB1, LEF1, AR, and TFE3 are closely interrelated with each other.

In this study, we aimed to identify useful biomarkers for predicting the malignant behavior of SPN. There remains a lack of consensus of the criteria for “malignant” SPN. The concerning features, including perineural invasion, vascular invasion, or deep infiltrative growth, that are commonly found in other malignant neoplasms do not indicate malignant behavior in SPNs [1]. Consequently, all SPNs are presently classified as low-grade malignant neoplasms. For these reasons, we considered distant metastasis and recurrence as decisive malignant features of SPN. Several studies have also failed to demonstrate significant impacts of certain features that can predict metastasis or recurrence; however, an undifferentiated carcinoma component, a larger tumor size (> 8cm), microscopic malignant features (such as cellular atypia), pleomorphism, capsule invasion, peripancreatic tissue invasion, perineural invasion, lymphovascular invasion, and a high mitotic rate have all been suggested as risk factors for recurrence [12, 14-16]. Despite this, we could not find any correlation between metastasis and clinicopathologic parameters, including nuclear pleomorphism, mitotic count, perineural invasion, peripancreatic extension, an infiltrative tumor border, and our putative biomarkers. For recurrence, a larger tumor size (> 5cm) was the only correlated factor. We evaluated the association between our immunohistochemical results and the malignant behavior of SPN, and found no relationship. We also performed Ki-67 immunohistochemistry and showed that SPNs with a KI-67 proliferative index of more than 5% were significantly associated with malignant behavior. A high proliferative index using Ki-67 immunohistochemistry has been associated with aggressive biological behavior of SPNs [14, 15]. We therefore suggest that close follow-up is needed in patients with a Ki-67 proliferative index of more than 5%.

In summary, we found that LEF1, TFE3, and AR were putative diagnostic markers of SPN auxiliary to β-catenin. Incorporated into an immunohistochemical panel, they could be beneficial to distinguish SPN from PDC and NET. In addition, we suggest that the Ki-67 proliferative index could be a predictive marker of metastasis in SPN.

## MATERIALS AND METHODS

### Case selection

Ninety-one formalin-fixed, paraffin-embedded (FFPE) specimens of SPN were obtained from the archives of the Department of Pathology, Yonsei University, Seoul, Korea and from the Liver Cancer Specimen Bank of the National Research Resource Bank Program of the Korean Science and Engineering Foundation of the Ministry of Science and Technology; the cases were encountered between 1999 and 2016. From consecutive cases of surgically resected pancreases, 51 PDCs were retrieved from 2009 to 2011 and 48 NETs were obtained from 2009 to 2015. Hematoxylin and eosin (H&E)-stained slides of all cases were reviewed. Histologic diagnosis of SPN was made based on microscopic features and one or more immunohistochemical markers including beta-catenin, CD10, E-cadherin, and chromogranin [1].

### Tissue microarray construction

Ninety-one cases of SPN, 51 of PDC, and 48 of NET were used for tissue microarray (TMA) construction using FFPE samples. A representative area of each case was selected under the microscope. Core tissues (3 mm in diameter) were taken from the individual FFPE blocks (donor blocks) and arranged in recipient paraffin blocks (tissue array blocks) using a trephine apparatus. Thirty-eight SPNs were selected and examined using conventional whole sections to rule out intra-tumoral heterogeneity.

### Antibody selection and immunohistochemistry

In our previous study, we found 2,026 up-regulated genes in SPNs compared with non-neoplastic pancreas. Among them, 1,119 genes were exclusively up-regulated in SPNs compared to PDCs and NETs. Beta-catenin, AR and WNT inhibitory factor 1 (WIF-1) have been identified by western blotting and immunohistochemical analysis [6]. Beta-catenin and fused in sarcoma (FUS) have also been identified as overexpressed proteins in our previous proteome expression profiling study of SPN [8]. We chose lymphoid enhancer-binding factor 1 (LEF1) and transcription factor for immunoglobulin heavy-chain enhancer 3 (TFE3), which were associated with Wnt signaling pathway. Therefore, immunohistochemistry was performed with six commercially available antibodies to these proteins (Beta-catenin, AR, WIF-1, FUS, LEF1, and TFE3).

Immunohistochemistry was conducted on 4-μm TMA tissue sections by a Ventana Bench Mark XT Autostainer (Ventana Medical Systems, Tucson, AZ, USA). Details of the tested primary antibodies are shown in Supplementary Table 1. The pattern of immunohistochemical staining was examined by intensity and area (%) as follows; negative (no staining or staining of less than 5% of tumor cells), weak positive (faint protein expression in 5%–30% of tumor cells), moderate positive (faint protein expression in > 30% of tumor cells or definite protein expression in less than 30% of tumor cells), and strong positive (definite protein expression in > 30% of tumor cells) [9, 20, 24].

We also evaluated Ki-67 proliferative index using whole section slides to investigate its associations with prognosis. For calculating Ki-67 proliferative index (Ki-67 positive tumor cells/total counted tumor cells, %), each tumor slide was manually scanned with a microscope at × 20 objective, and the area of greatest Ki67 positivity (hot spot) was selected for photography. Colored image of the hot spot was captured, and Ki67-negative and -positive tumor cells were marked in different colors. At least 500 tumor cells were counted. Pale staining nuclei were ignored during counting [33].

### Statistical analyses

Comparison of qualitative variables between groups was performed using the chi-square or Fisher’s exact test. The diagnostic sensitivity and the specificity for each of the markers individually, as well as in different combinations, were calculated. Using a logistic regression model, univariate analysis was conducted and odds ratios were estimated to assess predictive markers for distant metastasis. Two-sided P-values of less than 0.05 were considered statistically significant. Statistical analyses were performed using IBM SPSS 22 software for Windows (IBM Corp, New York, USA).

## SUPPLEMENTARY MATERIALS TABLES



## Abbreviations

AR: Androgen receptor
FFPE: formalin-fixed, paraffin-embedded
FUS: fused in sarcoma
H&E: Hematoxylin and eosin
LEF1: Lymphoid enhancer-binding factor 1
NET: neuroendocrine tumor
OR: odds ratio
PDC: pancreatic ductal carcinoma
SPN: Solid pseudopapillary neoplasm
TFE3: Transcription factor for immunoglobulin heavy-chain enhancer 3
TMA: tissue microarray
WHO: World Health Organization
WIF-1: WNT inhibitory factor 1
