# Supplementary material for: LEF1, TFE3, and AR are putative diagnostic markers of solid pseudopapillary neoplasms
Source: Oncotarget. 2017 Oct 16;8(55):93404–13. doi: 10.18632/oncotarget.21854 (PMC5706805; doi:10.18632/oncotarget.21854)
Supplement: Supplementary file 1 [file oncotarget-08-93404-s001.pdf]

LEF1, TFE3, and AR are putative diagnostic markers of solid pseudopapillary neoplasms

SUPPLEMENTARY MATERIALS

Supplementary Table 1: Primary antibodies and conditions

Marker	Clone	Dilution	Type of antibody	Source
Beta-catenin	14/Beta-Catenin	1:200	Mousemonoclonal	BD Biosciences, San Jose, CA, USA
AR	SP107	Prediluted	Rabbit monoclonal	Roche, Mannheim, Germany
LEF1	EPR2029Y	1:100	Rabbit monoclonal	Abcam, Cambridge, MA, USA
TFE3	MRQ-37	1:100	Rabbit monoclonal	Cell Marque, Rocklin, CA, USA
FUS	11570-1-AP	1:100	Rabbit polyclonal	Proteintech Group, Inc., Chicago, IL, USA
WIF-1	Sc373780	1:30	Mousemonoclonal	Santa Cruz Biotechnology, Santa Cruz, CA, USA
Ki-67	MIB-1	1:130	Mouse monoclonal	DAKO, Glostrup, Denmark

Supplementary Table 2: Clinicopathologic characteristics of 91 SPNs

		Sex		
	**Total, n (%)**	**Male**	**Female**	**P-value**
	91 (100)	16 (17.6)	75 (82.4)	
Age (y)				
≤ 40	61 (67.0)	8 (53.3)	53 (69.7)	0.240
> 40	30 (33.0)	7 (46.7)	23 (30.3)	
Size (cm)				
0-5.0	58 (63.7)	11 (68.8)	47 (62.7)	0.451
5.1-10.0	27 (29.7)	4 (25)	23 (30.7)	
>10.0	6 (6.6)	1 (6.3)	5 (6.7)	
Extensive hemorrhage/necrosis	25 (27.5)	1 (6.3)	24 (32)	0.029
Pleomorphism	6 (6.6)	3 (18.8)	3 (4.0)	0.065
Infiltrative tumor border	73 (80.2)	16 (100)	57 (76)	0.035
Peripancreatic invasion	40 (44)	12 (75)	28 (37.3)	0.011
Lymphovascular invasion	3 (3.3)	1 (6.3)	2 (2.7)	0.444
Perineural invasion	47 (51.6)	11 (68.8)	36 (48)	0.172
Lymph node metastasis	3 (3.3)	2 (12.5)	1 (1.3)	0.079
Distant metastasis	4 (4.4)	1 (6.3)	3 (3.9)	0.575
Recurrence	3 (3.3)	1 (6.3)	2 (2.7)	0.472
